# Do Task Sets Compete in the Stroop Task and Other Selective Attention Paradigms?

**DOI:** 10.5334/joc.272

**Published:** 2023-05-04

**Authors:** Benjamin A. Parris, Nabil Hasshim, Ludovic Ferrand, Maria Augustinova

**Affiliations:** 1 Department of Psychology, Bournemouth University, UK; 2 School of Applied Social Sciences, De Montfort University, Leicester, UK; 3 UniversitéClermont Auvergne, CNRS, LAPSCO, 63000 Clermont-Ferrand, France; 4 Normandie Université, UNIROUEN, CRFDP, 76000 Rouen, France

**Keywords:** task sets, Stroop task, interference, task conflict, phonological processing

## Abstract

Task sets have been argued to play an important role in cognition, giving rise to the notions of needing to switch between active task sets and to control competing task sets in selective attention tasks. For example, it has been argued that Stroop interference results from two categories of conflict: *informational* and *task (set)* conflict. Informational conflict arises from processing the word and is resolved by a late selection mechanism; task conflict arises when two task sets (i.e., word reading and colour identification) compete for activation and can be construed as involving an early selection mechanism. However, recent work has argued that task set control might not be needed to explain all of the switching cost in task switching studies. Here we consider whether task conflict plays a role in selective attention tasks. In particular, we consider whether S-R associations, which lead to informational conflict, are enough on their own to explain findings attributed to task set conflict. We review and critically evaluate both the findings that provided the original impetus for proposing task conflict in selective attention tasks and more recent findings reporting negative facilitation (longer RTs to congruent than to neutral stimuli) – a unique marker of task conflict. We then provide a tentative alternative account of negative facilitation based on poor control over informational conflict and apply it to a number of paradigms including the Colour-Object interference and Affordances tasks. It is argued that invoking competition between task sets in selective attention tasks might not be necessary.

A *task set* has been defined as a collection of control settings or task parameters that program the system to perform processes such as stimulus identification, response selection, and response execution (Vandierendonck, Liefooghe, & Verbruggen, 2010). Said differently, to adopt a task-set is to select, link, and configure the elements of a chain of processes that will accomplish a task (Rogers & Monsell, 1995). Task sets and their control have been argued to play an important role in cognition, giving rise to the notions of needing to switch between two or more intentionally activated task sets in task switching tasks (Monsell, 2003; Rogers & Monsell, 1995) and needing to control conflict between an intentionally activated task set and a competing, exogenously activated, irrelevant task set in selective attention tasks (known as *task conflict*; Goldfarb & Henik, 2007; Kalanthroff et al., 2018; Monsell, Taylor & Murphy, 2001; Parris et al., 2022).

Recently, however, the extent to which task sets and their control determine cognitive performance has been questioned. In the context of task switching, processing costs that were previously attributed to controlled switching between active task sets, have been accounted for with reference to feature-integration biases (see Schmidt, Liefooghe & DeHouwer, 2020). In parallel, several recent accounts have questioned the attribution of behavioural effects in selective attention tasks to cognitive control (Algom, Fitousi, & Chajut, 2022; Algom & Chajut, 2019; Schmidt, 2019), again questioning the need for cognitive control in cognition. Such arguments also pose a challenge to the foundational basis of the notion of task set competition and control in tasks of selective attention, especially given that the competing task set is exogenously, and not intentionally, activated in selective attention tasks.

We, amongst others, have been strong proponents of task conflict as a contributor to Stroop task performance and selective attention more generally (Augustinova et al., 2018; Augustinova, Ferrand & Parris, 2019; Ferrand et al., 2020; Parris 2014; see in particular Parris et al., 2022). Responses to Schmidt et al.’s (2020) model have also pointed out that feature-integration approaches fail to account for preparation effects in task switching studies, one of the key pieces of evidence for task set control in cognition (Monsell & McLaren, 2020; see also Koch & Lavric, 2020). Nevertheless, these recent developments in the wider fields of task set switching (Schmidt et al., 2020) and cognitive control (Algom et al., 2022; Algom & Chajut, 2019; Schmidt, 2019), and our own endeavours in questioning the evidence for the presence of conflict at other levels of processing (Burca et al., 2021; 2022; Hasshim & Parris; 2014; 2015; 2018; Parris et al., 2022), prompted us to reassess the role that task conflict and its control play in selective attention paradigms.

To be clear, our aim is not to argue that task sets do not play a role in selective attention tasks. The intentional, goal-oriented task set of colour naming seems to be a pre-requisite to achieve the instructed goal of naming the colour of the font in the Stroop task. Without it, what would participants do on presentation of this unusual stimulus? They would ignore the font colour and likely read the word – or they would just get up out of their chair and walk away. Our aim is not therefore to question the functional role of intentionally activated task sets, but is instead to question the role of exogenously activated task sets in selective attention tasks. Among the questions that could be asked regarding the exogenously activated task set is which task set becomes activated? Since most objects afford a number of tasks (e.g., a word can be read, categorised, defined, counted) which task set is exogenously activated when that object is presented? In the context of task switching, it seems reasonable to assume that it is the most recently activated task set, which is defined by the experimental context. But in selective attention tasks it is not so clear. Moreover, it is not clear what properties of an irrelevant stimulus activate a task set. Proponents of task set conflict in selective attention tasks would argue that it is “word-likeness” that exogenously activates the task set for reading but it is unclear what properties define word-likeness, nor is it clear when this happens and whether S-R associations without a task set guiding them such as orthographic to phonological connections or phonology to phonetic code connections are themselves enough to account for the effects observed without resorting to the notion of the whole task set for word reading (or whole set of S-R associations) being activated in advance of their execution.

We aim to go beyond recent reviews of task conflict (Littman, Keha, & Kalanthroff, 2019; Parris et al., 2022) by taking a broader and more critical look at the role of exogenously activated task sets. Moreover, in order to buttress our critical evaluation, we aimed to provide a tentative alternative account of the data – one based on established S-R associations that provide words with privileged access to word forms, and one that draws on some of the weaknesses of current task conflict theory – to highlight the notion that other accounts of the data are at least feasible. Indeed, in our recent review of levels of conflict and facilitation in the Stroop task (Parris et al., 2022), we concluded that whilst there was strong evidence in favour of the notion of task conflict in the Stroop task, more work was needed to identify what triggers activation of the task set for word reading and how other types of conflict, particularly phonological conflict, might interact with task conflict. We take this as our starting point but, for the purposes of testing the task conflict framework, go further by adopting the perspective that S-R associations can account for all effects attributed to task conflict. Whilst we focus here mainly on the Stroop task (Stroop, 1935) – since, to date, this is where most of the work on the role of task conflict in selective attention has been done – recent investigations have provided evidence for the influence of task conflict in other selective attention paradigms (e.g., the Affordances Task – see Littman & Kalanthroff, 2020; 2022) and thus, we also evaluate the evidence for task conflict in these paradigms. Our endeavour is therefore relevant to the wider field of selective attention and cognitive control.

## Distinguishing Task conflict from Informational conflict

Here we follow Rogers and Monsell (1995) in accepting that defining what constitutes a ‘task’, is, to use a common phrase that serves to highlight the point being made, a ‘difficult task’. We will therefore jump right past this definitional problem and “leave this conceptual boundary cloudy” (Rogers & Monsell, 1995: 208) without fear that doing so will impede our aims and objectives.

We can however offer a definition of task conflict. For task conflict to occur, at least two task sets must compete for activation. Given our definition of a task set above, this means that the entire collection of control settings/task parameters that program the system to perform one task (e.g., word reading consisting of visual analysis, letter/grapheme identification, lexical identification (semantic processing), phonological processing) would compete for activation with the entire collection of control settings that program the system to perform another task (e.g., classify a colour consisting of visual analysis, colour identification, semantic processing, phonetic encoding). The idea is that “whole task sets compete, over and above any competition between specific responses associated with a stimulus” (Monsell et al., 2001: 139–140). That is, over and above any S-R associations. In the context of the Stroop task, “task conflict” has been evidenced in three main ways: 1) Anterior cingulate activation on congruent trials (MacLeod & McDonald, 2000); 2) slower responses to neutral words (e.g., the word ‘house’) than to non-lexical (i.e., non-pronounceable) stimuli (e.g., ‘xxxx’; see Augustinova, Parris & Ferrand, 2019); 3) slower responses to congruent stimuli than to non-lexical stimuli, also known as reverse or negative facilitation (e.g., Goldfarb & Henik, 2007).

Representations that are activated by S-R associations (e.g., grapheme-to-phoneme conversion; or, for an irregular word, the whole orthographic form and the whole phonological form), and the representations/outputs produced by task sets (e.g., the name of a colour), can be described as *information*. Information derived from processing one stimulus, or one dimension of a stimulus (e.g., semantic information), can compete with information derived from processing another stimulus or another dimension of the same stimulus; this is called *informational* conflict. Thus, task conflict can be distinguished from the conflict that arises from the information that results from the operation of task sets or from S-R associations. Therefore, conflict between task sets cannot be phonological conflict, since phonological or phonetic (word form) representations are outputs and not whole task sets.^1^ Likewise, task set conflict is also not equivalent to the *semantic conflict* that might occur due to both the colour and the word dimensions of colour-word Stroop stimuli being processed at the semantic level. Neither is task set conflict *response conflict*, which results from the activation of potential response options given the prior information accrued from earlier processing of both dimensions of the Stroop stimulus. In short, under the task conflict framework, task conflict is independent of informational conflict, although informational conflict would not be entirely independent of task conflict given that some information is assumed to result from the operation of task sets. Clearly, it would be helpful for the purposes of distinction that, given its theorised independence, task conflict could be measured using a unique performance marker. Task conflict theorists have provided one such measure in the form of reverse or *negative facilitation* – the finding that congruent trial RTs can be *longer* than a neutral, non-lexical baseline (e.g., xxxx) in conditions described as a low task conflict control context. One aim of the present paper is to provide a tentative alternative account of negative facilitation; one that does not rely on invoking competition between task sets. In our alternative account, we consider whether negative facilitation could result purely from the notion that words have privileged access to word forms via strong S-R associations (e.g., Mahon, Costa, Peterson, Vargas & Caramazza, 2007; Roelofs, 2003).

## Outline of the paper

In the present paper, we set out to provide a reassessment of the task conflict framework as applied to selective attention tasks. Importantly, our aim was not to unequivocally argue that task conflict does not occur; it was instead to highlight current limitations of the account and to assess how much can be explained without it. To achieve this aim, we will: 1) describe and evaluate the studies and findings that provided the initial impetus for suggesting the influence of task conflict in selective attention tasks; 2) review the evidence for the more recent phenomenon of negative facilitation (congruent > non-lexical neutral), purportedly a unique marker of task conflict; 3) describe what we see as current challenges to the task conflict framework; 4) provide a tentative, alternative, and testable account of negative facilitation based on the notion of reduced control over S-R associations that results from certain experimental contexts; 5) discuss negative facilitation in the context of other selective attention tasks and how the alternative account might explain findings in those contexts.

## 1. Task and Informational conflict in the Stroop task

The Stroop task requires participants to focus on one dimension of a stimulus, the colour dimension, whilst ignoring another dimension, the word dimension. The task produces the Stroop *interference* effect – referring to the fact that identifying the colour that a word is printed in takes longer when the word denotes a different colour (colour-incongruent trials; e.g., the word *red* displayed in blue font) compared to a baseline condition (colour-neutral trials; e.g., the word *top* displayed in blue font). In addition, words that are congruent with the colour (colour-congruent trials; e.g., the word red in red font) result in faster colour-identification times when compared to a neutral baseline condition, producing Stroop *facilitation* effect (Dalrymple-Alford, 1972; Dalrymple-Alford & Budayr, 1966; Hasshim & Parris, 2021; see also MacLeod, 1991, for a review).

The extent to which the information generated from the irrelevant dimension (e.g., phonological, semantic and response information) *differs* from that generated from the relevant dimension was thought to determine the degree of Stroop interference that is subsequently observed (Klein, 1964). When considering the standard incongruent Stroop trial where the word dimension is a colour word that is incongruent with the target colour dimension that is being named (e.g., *red* in blue), and where the colour red is also a potential response, one might surmise numerous levels of representation where information from these two dimensions might compete. Processing of the colour dimension of a Stroop stimulus in order to name the colour would, on a simple analysis, require initial visual processing to establish colour identification, followed by activation of the relevant semantic representation and then word-form (phonetic) encoding of the colour name in preparation for a response. For this process to advance unimpeded until response there would need to be no competing representations activated at any of those stages.

Like colour naming, the processes of word reading also requires visual processing but of letters and not of colours. Word reading also requires the computation of phonology from orthography which colour processing does not. Despite being a task in which participants do not intend to engage, stimulus-response (S-R) associations mean irrelevant words are obligatorily processed (Monsell et al., 2001). Until recently, the evidence for conflict at the level of semantics was confounded (Parris et al., 2022). However, there is now good evidence for competition at the level of semantics (Burca et al., 2021; 2022). Likewise, obligatory phonological processing leads to obligatory phonetic encoding giving rise to competition between word form representations (Coltheart et al., 1999; Marmurek, Proctor & Javor, 2006; Parris et al., 2019). Finally, since both dimensions give evidence towards different responses, there is competition at the level of the response effectors (e.g., actual pronunciation with a vocal response Stroop task and finger selection with a manual button-press response). Importantly there is evidence that even when participants respond with a manual response the irrelevant word is pronounced sub-vocally (e.g., Parris et al., 2019).

For most of their history, Stroop interference and facilitation were thought to result from the information conveyed by the irrelevant word dimension that competed with that computed from the target dimension at late selection stages. However, the introduction of the notion of task conflict (Goldfarb & Henik, 2007; MacLeod & MacDonald, 2000; Monsell et al., 2001) changed that perspective indicating an earlier selection mechanism that controlled activation of the whole task set of word reading even before information was generated from that word. Motivated by the finding that the anterior cingulate cortex (ACC, i.e., a neural structure thought to be involved in monitoring for conflict; Botvinick et al., 2001), had been reported to be more activated by colour-congruent (and colour-incongruent) stimuli than a non-lexical neutral condition (e.g., Bench et al., 1993), MacLeod and MacDonald (2000) argued that the ACC activity represented the need to direct attention away from words irrespective of their congruency status, producing a form of interference present even on colour-congruent trials.

It was Monsell and colleagues (2001) however who were the first to attribute some of the interference in the Stroop task directly to task conflict. They argued that if Stroop interference resulted purely from informational conflict, it should be greater for irrelevant non-colour related words that have stronger connections to their associated lexical information (Cohen, Dunbar & McClelland, 1990; e.g., high frequency words), since their associated information would be accessed more readily. In other words, the word forms of high frequency words would be activated more quickly and would delay the production of the word form of the colour name when compared to low-frequency words. Or real words should be coloured named more slowly than pronounceable non-words. In their influential study Monsell and colleagues reported that interference was not larger for items that are more efficiently read (e.g., high vs. low frequency words, neutral words vs. pseudowords) and concluded that informational conflict (i.e., response conflict) is unable to account for the finding of largely undifferentiated interference produced by these colour-neutral Stroop items. Their results were inconsistent with predictions from Cohen et al. (1990) and, indeed, Monsell et al. actually reported that, if anything, colour naming times were slower for low frequency, not high frequency, items in their experiments.

Given the evidence for a role for task sets in cognitive processing (Monsell, 2003; Rogers & Monsell, 1995), Monsell et al. (2001) argued that their findings were best explained as resulting from competition between task sets; the endogenously activated task set for colour naming and the exogenously activated task set for word reading. This unintentionally activated word reading task set, competes with the intentionally activated colour identification task set, creating task conflict. Thus, they argued that, in addition to informational conflict, Stroop effects derive from task conflict. Monsell et al. suggested that the construct of task set selection could be construed as an “early selection” mechanism that filters irrelevant information. In support of this, see Hershman & Henik (2019; 2020) for pupillometric evidence for task conflict occurring earlier in the response time distribution than informational conflict in the Stroop task (although see below for an alternative interpretation).

Task conflict has been argued to be present in the Stroop task whenever an orthographically plausible letter string is presented (e.g., the word *table* leads to interference, as does the non-word but pronounceable letter string *fanit*; the letter string *xxxxx* less so; Levin & Tzelgov, 2016; Monsell et al., 2001). Accordingly, Monsell et al. suggested that the word reading task set is exogenously activated by properties of a stimulus that make it word-like such as patterns of consonants and vowels arranged in an orthographic structure (Taft, 1979; 1987) or by pronounceability, activated by sub-lexical orthographic to phonological connections.

Both MacLeod and MacDonald (2000) and Monsell et al. (2001)’s arguments provided the foundational bases for subsequent research into task conflict in the Stroop task. However, recent investigations invite a reconsideration of their interpretations of the findings. Stroop task performance has been shown to be unaffected following damage to dorsal ACC (Fellows & Farah, 2005). Furthermore, ACC activity has been argued to be dependent on the trial types (e.g., incongruent, congruent, repeated letter string) being presented intermixed in the same block; when presented in pure bocks (i.e., just incongruent trials), ACC activity does not differentiate between trial types (Floden, Vallesi & Stuss, 2011; see also Parris et al., 2019). Notably, two of the three studies cited by MacLeod and MacDonald as evidencing ACC activity on congruent trials (e.g., Carter, Mintun & Cohen, 1995; Milham et al., 2002; but see Bench et al., 1993) intermixed congruent and neutral trials, indicating that ACC activation is not necessarily a result of conflict on single Stroop trials but represents some other factor that is present when trial types are mixed.

In findings that also encourage a reinterpretation of one of the original motivations for task conflict, there is now ample evidence that Stroop interference is related to item naming efficiency, but where words that are named more slowly are also colour-named more slowly (Algom, Chajut & Lev, 2004; Burt, 1994; Burt, 1999; Burt, 2002). Whilst Monsell et al. were hesitant to make much of their finding showing that low frequency words were colour named more slowly than high-frequency words (because the effect was “small, its reliability was marginal”, p148), the effect has subsequently been repeatedly reported in the literature indicating that much more should be made of it (Burt, 1994; Burt, 1999; Burt, 2002; Dewhurst & Barry, 2006; Levin & Tzelgov, 2016; Navarrete et al., 2015). It is also notable that this effect has been replicated in the picture-word interference task, with pictures presented with low frequency distractors named slower than those with high frequency distractors (Miozzo & Caramazza, 2003; Dhooge & Hartsuiker, 2010).

These findings suggest that in Stroop-like tasks, target naming does not happen until the word is processed, representing a complete failure of selective attention in the context of the neutral-word Stroop task. This is then indicative of an irrelevant word form (i.e., the word name) interfering with the production of the relevant word form (i.e., colour name), representing a competition between responses, even if the irrelevant response is not a valid response for the task. Moreover, the finding indicates that the complete phonological or orthographic code is required before colour naming can occur. Thus, for neutral, non-colour related irrelevant words the extent to which lexical processing interferes in responding to the relevant colour dimension is directly influenced by word processing efficiency (e.g., attributes such as frequency, readability, pronounceability).

Monsell et al. proffered an explanation for the frequency effect, stating that it might result from the occasional “breakthrough” of the word when words are sufficiently primed and participants therefore fail to suppress the word reading task set. This would then create competition at the response selection stage resulting in the need for the conflict to be dealt with. This occasional generation of an inappropriate response would happen more quickly for high-frequency words and be sooner dealt with, resulting in short RTs for high-frequency words. However, an alternative account exists that does not rely on the occasional breakthrough argument, nor on the early selective filtering role of task set selection.

Under the Response Exclusion Hypothesis (Mahon et al., 2007) irrelevant words get obligatorily processed right up to the point of a representation entering an articulatory buffer; no selection occurs before this very late point in processing and selection does not involve selection by competition. Under this account words have privileged access to the articulators; thus, as with Roelofs (2003) and Glaser and Glaser (1989) this model is based on architectural differences between word reading/naming and colour naming. They describe this privileged access as being based on the “quasi rule-like relationship between orthography and phonology” (p. 524; Mahon et al., 2007) and as such results in a “production-ready” representation for the articulators to produce. The response exclusion hypothesis uniquely predicts that low frequency words should interfere more than high frequency words, a finding that theories, based on connectionist architecture (Cohen et al., 1990), find difficult to explain. Under this account frequency effects arise because of the principle that the earlier the response to the distractor enters the articulatory buffer, the earlier it can be removed from the buffer. Since low frequency words would take longer to reach the buffer, it takes longer to remove them from the buffer and thus colour naming times would be slowed. Thus, whilst they differ in their positions about the loci/locus and mechanism of selection, both Monsell et al. and Mahon et al. argue that slower responses for low frequency irrelevant words result from the fact that information about low frequency items take longer to be generated and to be later dealt with.

In our alternative account, we will argue that obligatory word form (phonetic) encoding of pronounceable letter strings (resulting from strong S-R associations (accrued from when children start to learn to read; Roelofs, 2003) that ultimately lead to words having privileged access to pronunciations) leads to interference from pronounceable letter strings due to the delaying of the retrieval of the pronunciation of the target colour name. This delay would not occur for letter strings such as xxxx because they are not pronounceable, but would occur for congruent words thereby producing negative facilitation (congruent RTs > non-lexical neutral RTs) in certain contexts (see below for an account of why any positive facilitation from congruent words does not counteract negative facilitation). In contrast to task set selection, this is a late selection mechanism. Moreover, it will be argued that this obligatory encoding is made more influential by experimental manipulations, purportedly used to induce task conflict, but that actually result in less control over informational conflict – the phonological and phonetic encoding of the irrelevant word – making it harder to inhibit, and that prevent positive facilitation. Thus, it is the combination of poorer control over informational conflict, and the non-pronounceable nature of the baseline (e.g., xxxx), that produces negative facilitation. We will argue that this occurs even with manual-response Stroop tasks for which phonological processing is reduced compared to vocal response Stroop tasks and with which most of the effects of negative facilitation have been reported.

## 2. The Stroop task and Negative Facilitation – the prime behavioural marker of task conflict

### 2.1. Task conflict in a mostly non-lexical trial context

As already mentioned, a common finding in the Stroop literature is that colour-identification is faster for colour-congruent than for neutral non-lexical stimuli (Brown, 2011; Entel et al., 2015; see also Augustinova et al., 2019; Levin & Tzelgov, 2016, Shichel & Tzelgov, 2018) – also known as positive facilitation. If, according to task conflict approach, congruent trials involve task conflict but non-lexical stimuli do not (see MacLeod & MacDonald, 2000; Monsell et al., 2001 discussed above), why is *negative facilitation* (i.e., slower responses to congruent trials compared to non-linguistic baselines) not the more common finding? Task conflict theorists argue that positive facilitation is expressed only when sufficient task conflict control is activated.

Goldfarb and Henik (2007) reasoned that negative facilitation is often not observed when comparing congruent and non-lexical trial RTs because there is a sufficiently large enough proportion of lexical stimuli to activate task conflict control. In other words, constant exposure to real words puts the task conflict controller on high alert, ensuring that it is active, and that task conflict is kept low. The activation of task conflict control means that positive facilitation, representing a lack of another form of control over informational processing, is expressed in the RT data. Increasing the proportion of non-lexical neutral trials (e.g., repeated letter strings) would create the expectation for a low task conflict context thereby reducing task conflict control, resulting in task conflict’s unique behavioural expression – negative facilitation. Thus, Goldfarb and Henik (2007) introduced the notion of a task conflict controller that forms part of a system of cognitive control that is deployed to reduce or prevent task conflict (see also Kalanthroff et al., 2018; and Littman et al., 2019, for a mini review).

In line with this reasoning, Goldfarb and Henik (2007) aimed to reduce task conflict control by increasing the proportion of non-word neutral trials (repeated letter strings) to 75% (see also Kalanthroff et al., 2013a). Additionally, on half of the trials, the participants received cues that indicated whether the following stimulus would be a non-word or a colour word, giving another indication as to whether the mechanisms that control task conflict should be activated. For non-cued trials, when task conflict is high, RTs were slower for congruent trials than for non-lexical trials, producing a negative facilitation effect. Cueing that the upcoming trial involved a congruent or incongruent word led to substantially reduced RTs for both congruent (834ms vs. 942ms) and incongruent (1002ms vs. 1070ms) stimuli and eliminated negative facilitation. Goldfarb and Henik (2007) suggested that previous studies had not detected a negative facilitation effect because resolving task conflict for congruent stimuli when task conflict control is sufficiently active (i.e, when there are ~ 50%+ lexical trials) does not take long, and thus, the effects of positive facilitation were able to be expressed in response times – in other words, positive facilitation had hidden task conflict.

Subsequent work has further clarified the conditions required to observe negative facilitation: Entel and Tzelgov (2018) showed that presenting participants with non-lexical trials and congruent trials (but no incongruent trials) where the portion of the former was 75%, and without presenting cues of any kind, was not enough to reveal negative facilitation. They did however show that this experimental context did result in reduced positive facilitation relative to a mostly congruent block, indicating that a mostly non-lexical block does reduce the capacity of the congruent word to positively facilitate performance. In contrast, a subsequent experiment negative facilitation was reported after pre-exposing participants to a block of incongruent trials prior to completing the mostly non-lexical block and/or when including incongruent trials in a final block. Entel and Tzelgov (2018) explained this by suggesting that task control was not monitored in the first experiment due to the lack of exposure to incongruent trials, which is needed to generate control over the automatic reading process. This line of research suggests in sum that it is not cueing that is key to producing negative facilitation; an interpretation that is supported by the finding of no negative facilitation in Experiment 2 of Goldfarb & Henik (2007) in which non-lexical trials were replaced by neutral word trials but in which cueing was employed). Instead, a combination of mostly non-lexical trials and exposure to informational conflict are important in revealing large negative facilitation effects.

In contrast to the notion that informational conflict is necessary to observe negative facilitation, Kalanthroff et al. (2013a) reported negative facilitation when only non-lexical and congruent trials were included in a block and the stimuli were mostly neutral (i.e., the block only included trials that were free of informational conflict and of the response competition it entails). Unlike Entel and Tzelgov (2018), Kalanthroff et al. (2013a) did employ the same cueing procedure as that used in Goldfarb and Henik’s (2007) study. Again though, negative facilitation was observed in the non-cued block. However, as Entel and Tzelgov noted, Kalanthroff et al. (2013a)’s negative facilitation effect was relatively small (i.e., 15ms vs. the > 70ms reported by Entel and Tzelgov) and the Bayes Factor for the effect was below 3, indicating the evidence was anecdotal. Thus, the cueing context might add something to the production of negative facilitation, but it is the majority non-lexical trial context and exposure to incongruent trials that seems to be largely responsible for observing large negative facilitation effects.

Entel and Tzelgov (2020) followed up their previous work by showing that negative facilitation is not observed if participants’ working memory (WM) resources are taken up by a secondary task. They therefore argued that the expression of negative facilitation requires working memory, arguing that it indicated a role for proactive control. This later result directly contradicts the earlier findings of Kalanthroff et al. (2015) who showed that spare WM capacity prevents negative facilitation, indicating that proactive control plays a role in controlling the expression of task conflict – an idea captured in their computational model of task conflict (Kalanthroff et al., 2018; see here below). Entel and Tzelgov (2020) pointed out that the negative facilitation was again relatively small (i.e., 20 ms) in Kalanthroff et al.’s (2015) study, and that the Bayesian estimate of the support for it was anecdotal (BF_10_ = 2.48). In contrast, in Entel and Tzelgov’s study negative facilitation was large (~50ms) and was supported by a sensitive Bayes Factor. Thus, in contrast to Kalanthroff et al. (2018)’s model of task conflict (see the following section for description) the most robust evidence is currently consistent with the notion that negative facilitation requires spare working memory resources. However, given the contradictory nature of these findings strong conclusions cannot be drawn at this time regarding the role of working memory in producing negative facilitation.

To sum up, three factors that have been identified that contribute to observing negative facilitation RTs: 1) a mostly non-lexical trial context; 2) anticipation of informational conflict in the form of incongruent trials; 3) spare working memory capacity (Entel & Tzelgov, 2020; but see Kalanthroff et al., 2015; 2018). However, whilst exposure to incongruent trials and working memory capacity seem to play a role in modifying the magnitude of negative facilitation, the two studies investigating the role of working memory have found directly opposing results, and despite being small, significant negative facilitation has been reported without exposure to incongruent trials. Therefore, only the mostly non-lexical trial context and, most importantly, its use as the neutral baseline against which to compare congruent trial RTs, seems to be the *sine qua non* of significant negative facilitation effects in the studies reported above.

### 2.2. Negative facilitation without a mostly non-lexical trial context

Despite being important for producing negative facilitation in the above studies, negative facilitation has been reported in the absence of a mostly non-lexical trial context when the Stroop task has been combined with other paradigms. For example, negative facilitation has been reported in studies employing task-switching, the Stop-Signal task and when pupillometry is employed as the dependent variable.

#### 2.2.1. Negative facilitation and task-switching

If negative facilitation reflects task set conflict, it should be greatest when task conflict is at its height. In contrast to selective attention tasks, task switching tasks involve two intentionally activated task sets. For example, when the Stroop task is used in task switching studies, participants are required to switch between reading the word and naming the colour. This would mean that during colour naming trials, the task set for word reading would be more active than it would be in the context of a selective attention task. This would have the effect of increasing the relative conflict between the two task sets.

In two experiments Kalanthroff and Henik (2014) reported that negative facilitation was significant when the time between a cue indicating whether the upcoming task was word reading or colour naming and the appearance of the Stroop stimulus (the cue-target interval or CTI) was 0ms, but was not significant when CTI was 300ms or 1500ms. In other words, negative facilitation was larger when there was less time to prepare for the upcoming task (i.e., when task set competition was greater – similar to the results from Aron et al., 2004). There is also evidence of a relationship between negative facilitation and task-switching in studies that did not employ the Stroop task but did employ a neutral condition against which congruent trials could be compared (e.g., Aarts et al., 2009; Aron et al., 2004; Rogers & Monsell, 1995).

#### 2.2.2. Negative facilitation and the Stop-Signal task

Kalanthroff, Goldfarb and Henik (2013b) have shown that negative facilitation can be observed when the Stroop task is combined with the Stop-Signal Task even in conditions where the proportion of congruent, incongruent and repeated-letter trials are equal. In the Stop-Signal Task participants are asked to respond to particular stimuli (in this case the colour of the font of Stroop stimuli) unless a stop-signal is presented. In two experiments (1 and 3), the authors showed that on trials in which there was no stop signal (go trials), interference and positive facilitation were reported. However, on trials on which a stop-signal was presented but participants responded anyway (i.e., erroneous stop-signal response trials or ESSR), interference and negative facilitation were reported. Negative facilitation was not observed in their Experiment 2 in which the nonword neutrals were replaced with word neutrals. Kalanthroff and colleagues (2013b) argued that their results showed that task conflict and stop-signal inhibition share a common control mechanism, and one that is independent of the control mechanism activated by informational conflict – and this was in a context in which all trial types were presented equally often and when no other task conflict control manipulation was applied.

Kalanthroff et al. (2013b) argued that an ESSR can occur because (a) the primary go process was extremely fast or (b) the inhibitory control was momentarily less efficient and produced slower stopping, but favoured the latter position given that control is an effortful process. The authors also drew on neuroimaging results showing ACC activations on stop-signal trials (Brown & Braver, 2005; van Boxtel, van der Molen, & Jennings, 2005) to argue that the similarity in activations with nonneutral (incongruent and congruent) Stroop stimuli. They argued that negative facilitation is evident on ESSR trials because both task conflict and ESSR trials represent reduced inhibitory control. Furthermore, the authors replicated the finding showing that inhibition of initiated responses on stop trials were less likely and slower on incongruent Stroop trials (Verbruggen, Liefooghe, & Vandierendonck, 2004).

#### 2.2.3. Negative facilitation and pupillometry

Pupillometry has been shown to measure negative facilitation using the difference between colour-neutral words and non-lexical trials even in the absence of negative facilitation in the manual response RT data (Hershman & Henik, 2019; 2020). Using incongruent, congruent and a repeated letter string baseline, but without manipulating the task conflict context in a way that would produce negative facilitation (the proportion of trial types was equal and no cueing as employed), Hershman and Henik observed a standard Stroop interference effect and small non-significant, positive facilitation. However, the authors also recorded pupil dilations during task performance and reported both interference and negative facilitation in this metric (pupils were smaller for the repeated letter string condition than for congruent stimuli). The timeseries data showed that pupil data began to distinguish between the repeated letter string condition and incongruent and congruent conditions up to 500ms before there was divergence between the incongruent and congruent trials.

More recently, Hershman et al. (2021) reported that, in terms of colour naming response times, repeated letter strings did not differ relative to symbol strings (e.g., %&^$), consonant strings (e.g., CGHD) and colour-neutral words (e.g., table). All were responded to more slowly than congruent trials evidencing positive facilitation on congruent trials. However, they also reported that pupil size data revealed larger pupils to congruent trials compared to repeated letter strings, symbol strings, and consonant strings. This effect indicates that a letter string needs to be readable (pronounceable) for the task set for word reading to be activated and for task conflict to arise.

### 2.3. A model of task conflict

Prior to Entel and Tzelgov’s (2018; 2020) work, Kalanthroff et al. (2018) presented a model of Stroop task performance that successfully incorporates both task conflict and its control. This model is based on processing principles of Cohen and colleagues’ neural network models (Botvinick et al., 2001; Cohen et al., 1990) that describe the processing of stimuli as occurring via activation of a series of modules along two processing pathways – a word processing pathway and a colour processing pathway – with the possibility of each module being activated by each pathway simultaneously. When different pathways activate a common module in the output layer, this results in facilitation or interference depending on whether pathways activated by the word and colour dimensions are similar or different. For a congruent trial, facilitation results since both word and colour activate the module providing evidence for the same response in the output layer; in contrast, for an incongruent trial the two pathways provide evidence for different responses, resulting in interference. An important component of the model is that the momentary balance of evidence for each response is defined by the strength of evidence in favour of one minus the strength of evidence in favour of the other. When the difference between the two pieces of evidence crosses a threshold, selection occurs. To ensure that the correct response is produced a *Task Demand unit* biases processing to ensure the colour-processing pathway wins out. Therefore, although the biasing of attention toward a certain pathway (attentional selectivity) begins early in processing, its effect is to reduce competition at the response module to allow for more efficient response (action) selection.

What is unique about Kalanthroff et al. (2018)’s model is the role proactive (intentional, sustained; see Braver, 2012) and reactive control play in modifying task conflict. When proactive control is high, task conflict is low and thus negative facilitation is not present. When proactive control is low, task conflict is high, and an inhibition mechanism then operates to modify the response threshold to all lexical stimuli. Reactive control then operates to control task conflict but is not sufficient to prevent its behavioural manifestation (negative facilitation). This raising of the response threshold would not happen for repeated letter string trials (e.g., xxxx) because the task unit for word reading would not be activated. Since responses for congruent trials would be slowed relative to non-lexical trials, negative facilitation results. In contrast to Botvinick et al.’s (2001) model, reactive control is employed when proactive control is low, not when informational conflict is detected, and since task control can be activated even in the absence of informational conflict, task control is dissociable from informational conflict control (but see Entel & Tzelgov, 2020, who argue task conflict control is dependent on the detection of informational conflict). A further unique aspect of Kalanthroff et al.’s model is that it is able to account for the common finding of larger standard deviations for congruent than for non-lexical neutral trials, a finding that had been considered a weakness of Cohen et al.’s original model (see Mewhort, Braun & Heathcote, 1992). This increased variability was accounted for by task conflict interacting with noise on task demand units; thus, an additional level of conflict permitted the influence of an additional level of noise in the system.

## 3. Current challenges for the task conflict framework

Negative facilitation serves as a new and unique marker of performance that is independent of those findings. In this section we highlight some of the current challenges to the task conflict framework and its account of negative facilitation.

### 3.1. Is there a task selection problem?

Monsell et al. (2001) argued that the notion of “task set” is essential in explaining why we do not always name (or translate or classify) attentively fixated words. A written word, they argue, affords many possible tasks and thus the appropriate mental machinery needs to be pre-configured for the intended action to occur and the appropriate output to be produced. Recent research indicates that even for a process often considered ‘automatic’ – visual word recognition – certain aspects of word processing (such as word frequency and word/nonword status) can be delayed or attenuated following a previous non-word reading perceptual task (Elchlepp, Monsell & Lavric, 2021). However, if a fixated written word affords many possible tasks, which task set will be exogenously activated in selective attention tasks? In other words, is there a task selection problem? A task selection problem could lead to the exogenous activation of multiple relevant task sets (depending on recent intentionally activated task sets) resulting in tremendous task set competition. Nonetheless, it is also possible that given the activation of multiple task sets, no one task set is exogenously activated strongly enough to influence task performance; or perhaps, in selective attention tasks, they are not exogenously activated at all. Perhaps it is reasonable to assume that the word reading task set, as the more routinised task set, is the task set that is activated when a letter string is presented. However, why is the entire collection of control settings that program the system to perform reading (whole sets of S-R associations) needed in advance of reading when S-R association proceeding serially are enough for reading to happen.

### 3.2. Exogenous activation of task sets or just S-R associations?

**Figure 1:**
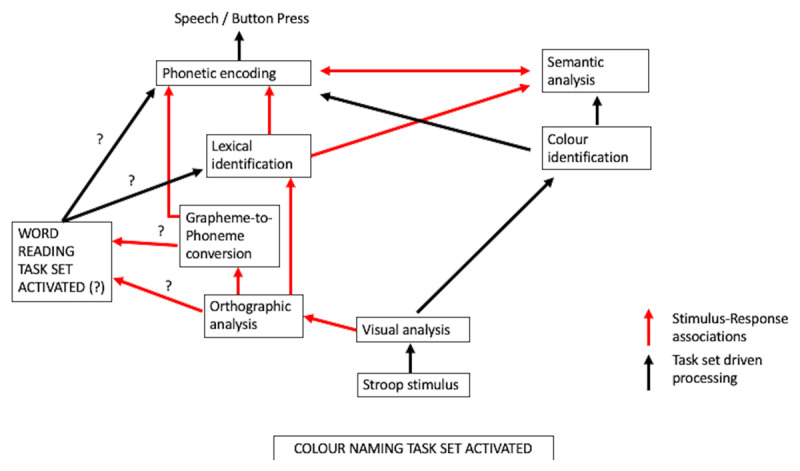
Modified version of the model presented in Monsell et al. (2001). What activates the task set for word reading and what does it go on to do? Are S-R associations such as grapheme-to-phoneme processing enough to explain the effects attributed to task conflict? An alternative account of negative facilitation is that strong S-R associations confer privileged access for pronounceable letter strings to their associated phonetic codes meaning that the irrelevant word is obligatorily processed up to the level of phonetic encoding, which delays phonetic encoding of the colour name. The longer the encoding of the irrelevant word takes, the longer the delay. For example, phonetic encoding is slower for low frequency words and thus colour naming is slower for such words compared to high frequency words. If this delay occurred for all pronounceable letter strings, but not for non-pronounceable letter strings, colour naming would be slower for all pronounceable letter strings leading to negative facilitation.

Alongside their novel argument that the Stroop task involves task set conflict, Monsell et al. (2001) noted that interference in the Stroop task also derives from S-R associations that automatically compute grapheme-to-phoneme correspondence rules that lead to response conflict (competition at the level of response output) – and potentially even process the word to the level of semantics in some contexts (e.g., Burca et al., 2021; 2022; Coltheart et al., 2001; Cohen et al., 1990; McClelland, & Rumelhart, 1981). These S-R associations do not need a task set directing their activation because they are themselves activated in response to certain stimuli (the presence of graphemes). Indeed Monsell et al. argued that these associations could be responsible for exogenously activating the task set for word reading given that sub-lexical orthographic to phonological connections give clues to pronounceability or the patterns of consonants and vowels arranged in an orthographic structure that confer word-likeness on a letter string (Taft, 1979; 1987). However, if S-R associations already lead to the processing of the irrelevant word, what is left for the task set for word reading to do (see Figure 1)? Is it that obligatory low-level processes (e.g., orthographic/phonological processing) represent the automatic S-R associations that trigger the task set for word reading (as argued by Levin & Tzelgov, 2016) which then leads activation of the task set for word reading and subsequent semantic and word form (phonetic encoding) processing? In this instance task conflict would influence performance relatively early in the processing stream. Or perhaps the S-R associations accomplish all of the above and the task set for word reading, linking as it does the elements necessary for a response, ensures the final response is guided by outputs from word reading processes. Without the task set for word reading perhaps the S-R associations would have no influence on performance. Another way of asking the same question is whether stimulus-driven behaviour necessitates invocation of exogenous task set activation? Could S-R associations be enough on their own? Do rules and settings need be preloaded to enable certain processes to run their course? And is it that all the to-be-used S-R associations need to be linked up in advance of processing or do they simply follow on one from the other in a serial manner without a guide for action? In the case of intended, planned actions, the notion of a task set aiding some aspects of goal-oriented behaviour, linking or strengthening the links between associated processing seems to have cognitive utility, but one might question whether it is a characteristic of unintended processing.

### 3.3. What is the effect of a mostly non-lexical trial context?

A long-standing and robust finding in the attentional control literature is that manipulations of the proportion of the different trial types modify Stroop effects (e.g., Kane & Engle, 2003). For example, Stroop interference is smaller when there are a greater proportion (e.g., 75%) of incongruent trials vs. congruent trials (see e.g., Lindsay & Jacoby, 1994; Logan & Zbrodoff, 1979). Accounts for this congruency proportion effect vary (see Braem et al., 2019, for a recent discussion). Within the selective attention domain, prominent theories often posit a *proactive control mechanism* (e.g., Botvinick et al., 2001; Braver, 2012) where the attention system is biased via a global, top-down manner, triggered by influences such as participants’ expectancies, motivation, and strategies. For example, in an environment where conflicting information (e.g., an incongruent Stroop trial) is often encountered, the conflict monitoring system will signal for more attentional resources to be utilised so as to aid task performance, in contrast to situations where conflict is not encountered as often, and the system does not get triggered. In contrast, Schmidt and Besner (2008) proposed a contingency learning explanation, driven by the fact that the frequencies of the colour-word pairs making up the Stroop stimuli are confounded by response contingencies in the classic PC paradigm. Under this account, a greater proportion of congruent trials leads to more Stroop interference because congruent words become strongly associated with their colour counterparts; that is, the word red predicts the response red. This response contingency is not present when there is a greater proportion of incongruent trials and hence the Stroop effect is smaller. More recently, Spinelli and Lupker (2021; 2022) reported that trial-type proportion manipulations can result in modified Stroop effects even when congruent trials are replaced by non-lexical trials (e.g., repeated-letter trials) trials. They showed that the Stroop effect is smaller, and thus proactive control is stronger, when there is a greater proportion of incongruent trials. Spinelli and Lupker argued that their data provided evidence for the operation of proactive control in a confound-free context. Importantly, for present purposes, their findings suggest that a mostly non-lexical trial context reduces proactive control.

Consistently, according to Kalanthroff et al. (2018)’s model the mostly non-lexical context induces a low task conflict control state, and thus reduces proactive control. When proactive control is low, task conflict is high, and an inhibition mechanism then operates to modify the response threshold to all lexical stimuli. This raising of the response threshold would not happen for repeated letter string trials (e.g., xxxx) because the task unit for word reading would not be activated. Since responses to congruent trials would be slowed relative to non-lexical trials, negative facilitation results. This would predict that the mostly non-lexical context affects all lexical stimuli equally and therefore that response, semantic and phonological conflict would be unaffected (provided a lexical baseline is used in the computation of their magnitudes).

In contrast, arguing from the perspective that positive facilitation results from inadvertent reading^2^ (Kane & Engle, 2003; MacLeod & MacDonald, 2000), Entel and Tzelgov (2020) reasoned that a larger number of non-lexical trials means that participants are less likely to inadvertently read the congruent word, thereby reducing facilitation and, presumably, reducing interference of all kinds.

Results from a recent study (Kinoshita, Mills, & Norris, 2018) put these interpretations of the effect of a mostly non-lexical context into doubt. They used the proportion of non-word trials manipulation to investigate whether semantic processing in the Stroop task can be controlled. They reasoned that they could manipulate the task of word reading by including a high proportion (i.e., 75%) of non-lexical trials (a rows of #s in this case). According to Entel and Tzelgov’s position, this should reduce semantic processing because a greater proportion of non-lexical trials reduces the likelihood of word reading. According to Kalanthroff et al.’s model, the response threshold for all lexical stimuli is raised by equal amounts suggesting that informational conflict would not be affected. In contrast to such predictions, Kinoshita and colleagues reported larger semantic Stroop effects (i.e., interference induced by colour incongruent items that were not in the set of response colours) despite using neutral words trials as the baseline against which semantic conflict was computed, in the condition with a high proportion of non-lexical trials. The authors argued the effect resulted from reduced control over word processing. This result contrasts with Entel and Tzelgov’s account of the effect of the mostly non-lexical trial context by showing that word processing was more likely in a mostly non-lexical context. It is also inconsistent with Kalanthroff et al.’s model which would predict that informational conflict should not have been affected. Their result is nevertheless consistent with the notion that a mostly non-lexical trial context reduces proactive control (Spinelli & Lupker, 2021; 2022). It is also worth noting that a mostly non-lexical trial context also increased informational conflict (incongruent – congruent) in studies that have included them (e.g., Goldfarb & Henik, 2007).

On the basis of this finding, it could be argued that large, robust negative facilitation effects are more likely to emerge in a mostly non-lexical trial context, not because of the effect it has on a task conflict control mechanism, but instead because it renders informational conflict control less effective leading to increased semantic, phonological and response processing of the irrelevant word. Nevertheless, Kinoshita et al.’s results need to be replicated before any strong conclusions can be drawn. Further studies investigating the effect of a mostly non-lexical trial context on varieties of informational conflict would be informative here.

### 3.4. Why is there a lack positive facilitation when task conflict control is supposed to be high?

As mentioned above, in their original study, Goldfarb and Henik (2007) reasoned that negative facilitation is not often found under standard Stroop task conditions because there is a sufficiently large enough proportion of lexical stimuli to activate task conflict control. The activation of task conflict control means that the resulting task conflict is minimal and positive facilitation can be therefore expressed in the RT data. If, however, the expectation for a low task conflict is introduced – via a greater proportion of non-lexical neutral trials – and task conflict control is thereby reduced, negative facilitation is observed in RTs. In line with this reasoning, substantial negative facilitation (i.e., ~130ms) was observed under these latter conditions in Experiment 1 of Goldfarb and Henik (2007)’s original study. However, in the control condition of this same experiment, in which the need for task control was cued and expected conflict and control was high, no positive facilitation was revealed, and a non-significant ~21ms of negative facilitation was actually observed. Similarly, in Experiment 2, which Goldfarb and Henik (2007) presented to show that there is no negative facilitation when task conflict control is increased (by swapping non-lexical neutral trials with neutral word trials ensuring the presence of task conflict on every trial), positive facilitation was absent and negative facilitation was present instead, albeit by a non-significant amount (~18ms).

This repeated absence of positive facilitation under conditions in which task conflict control should be high enough to expose it, is somewhat problematic for the task conflict account. The authors have in fact referred to negative facilitation as *reverse* facilitation, but there is no *reversal* of facilitation if, in the control condition, positive facilitation is clearly absent. Said differently, the finding of robust negative facilitation observed in Experiment 1 of Goldfarb and Henik’s original study, should be understood in light of null positive facilitation effects when positive facilitation effects are clearly expected (i.e., when no task conflict-related manipulations are applied).

The lack of positive facilitation seems to be a result of the mostly neutral trial context; whether that be non-lexical neutral or non-colour related word neutral trials. If, as suggested in the section above, we interpret the mostly neutral trial context as promoting reduced proactive control, the finding of no positive facilitation in a mostly neutral context would suggest that positive facilitation is itself the result of increased proactive control, which seems counterintuitive given that proactive control should lead to less, not more, word reading. However, given that positive facilitation is beneficial to performance it is conceivable that its expression is related to better control in some circumstances (as argued by the task conflict account).

If reduced proactive control results from a mostly non-lexical context relative to a mostly neutral word context and yet positive facilitation is absent even in a mostly neutral word context, then one might surmise that it is the lack of a mostly colour word context that impedes the expression of positive facilitation. It is clear that more research is needed to understand why positive facilitation is reduced in a mostly neutral trial context (and in other contexts we will go on to discuss such as the Stop-Signal task context). We do not intend to provide an account of this here since it is not directly addressed by the task conflict account of negative facilitation. Nevertheless, along with other trial type proportion effects, this seems to be an effect worthy of further investigation.

### 3.5. What is the role of proactive control/working memory?

Although related to the above two challenges it is worth highlighting the directly opposing arguments from task conflict theorists. Kalanthroff et al.’s (2018) model predicts that proactive control capacity leads to less negative facilitation. However, there exists robust evidence that working memory capacity is required for the expression of negative facilitation (Entel & Tzelgov, 2020). Entel and Tzelgov have interpreted this as showing that working memory capacity is required for proactive control. Thus, there are two directly opposing accounts of negative facilitation; one that argues that negative facilitation requires proactive control and the other that argues that negative facilitation results from poor proactive control.

## 4. Is there an alternative to a task conflict account of negative facilitation?

Above we have discussed challenges to the task conflict account of negative facilitation. However, it is important to note that there is currently no alternative account of negative facilitation in the literature. The finding that congruent trials result in longer response times than neutral trials in both selective attention and task switching contexts has been universally interpreted as resulting from competition between task sets. The lack of an alternative, testable account of negative facilitation perhaps attests to the power of the approach. Nevertheless, we set ourselves the challenge of providing an alternative and testable account of negative facilitation in the Stroop and other tasks in the hope that it will permit strong tests of the task conflict account. It is again worth pointing out that our aim is not to invite the reader to send the theoretical constructs of task set control and task conflict to the Occam’s Razor retirement home for superannuated theories, it is rather to encourage attempts to falsify it. Below we present a tentative alternative account that builds on some of the challenges presented above. Each of these sections outlines testable predictions based on this alternative to the task conflict account.

We will argue from the perspective that, in selective attention tasks, the irrelevant task set is not activated, or not activated enough, to influence performance and thus that whole task sets do not compete. We will argue that uncontrolled, obligatory (but not necessarily effortless) SR associations are sufficient to account for negative facilitation in most contexts. That is, a response strongly associated with a particular stimulus (including congruent stimuli) is activated independent of a participant’s goals whether that be the reflexive production of an object’s name on presentation of the object, the motoric response to the picture of an object with a graspable handle, or phonological, semantic or response processing of the irrelevant word in the Stroop task. Whilst we focus mainly on the Stroop task, the general argument can be applied to other tasks – and indeed we do so in later sections.

### 4.1. An alternative account of negative facilitation in the Stroop task

Our reading of the literature on negative facilitation in the Stroop task leads us to the conclusion that experimental manipulations intended to increase task conflict can also be thought of as manipulations that increase informational conflict. For example, according to the task conflict account, a mostly non-lexical trial context increases task conflict because it creates an experimental context in which task conflict control is not needed (mostly). This manipulation does seem to be important in producing negative facilitation. Indeed, outside of task-switching and stop-signal trial contexts it appears to be the only experimental manipulation that is able to produce a significant effect of negative facilitation (however small) by itself in the RT data, without any other manipulation such as cueing, low working memory load, or exposure to incongruent trials.^3^ However, a mostly non-lexical trial context has also been shown to increase semantic conflict (Kinoshita, De Wit & Norris, 2017 – see below), a form of informational conflict. In what follows we will outline an alternative of negative facilitation as reported in mostly non-lexical trial contexts, task-switching contexts and stop-signal task contexts – one based on the notion that it is informational conflict that causes negative facilitation, not competition between intentionally and unintentionally activated task sets. We start by considering the baseline against which congruent trials are often compared when negative facilitation is reported.

#### 4.1.1. A Pronounceability Cost

The observance of negative facilitation is determined by whether the baseline condition is non-lexical (e.g., repeated-letter strings) or lexical (colour-neutral words). As we have previously noted, selecting an appropriate baseline, and indeed an appropriate critical trial, to measure the specific component under test is non-trivial (Parris et al., 2022; see also Evans and Servant, 2022). Differences in performance between a critical trial and a control trial might be attributed to a specific variable but this method relies on having a suitable baseline that differs only in the specific component under test (Jonides & Mack, 1984). Nevertheless, task conflict proponents argue that a major difference between repeated-letter strings and colour neutral words is the presence of task conflict on neutral word trials and its absence on rows of Xs. Some researchers (including ourselves) have even used this difference as a measure of task conflict (Augustinova et al., 2019; Ferrand et al., 2020; Kinoshita et al., 2017; 2018). However, it has also been argued that the difference between these two trial types is a form of informational conflict called the *lexicality cost* resulting from the fact that a word “is known and has meaning” (Brown, 2011, p.86). Repeated-letter and other non-lexical baselines (e.g., a series of nonalphanumeric symbols) differ from lexical stimuli, including conflicting and congruent ones, in terms of this lexicality cost. It is therefore possible that negative facilitation appears, not because non-lexical trials lack task conflict, but because they lack a “lexicality” cost. However, Brown’s (2011) idea of lexicality was not coined to account for task conflict, and sounds somewhat similar to task conflict, albeit eschewing mention of competition between whole task sets. Also, and importantly, one can reasonably ask if there is any explanatory advantage of “lexical” conflict over “task” conflict since both involve the activation of the mental machinery for word reading. However, one big difference is that the latter case requires the selecting, linking, and configuring two sets of processes that accomplish different tasks, and for those collections of processes to compete independently of informational conflict. Moreover, S-R associations are required, at some point, to activate a task set. The former case requires simple S-R associations to proceed unbounded. However, in contrast to Brown’s approach we argue that Brown’s notion of a lexicality cost is best understood in terms of a *pronounceability cost*; a cost resulting from informational conflict, and is thus not defined as occurring when a word “is known and has meaning” (Brown, 2011: 86), but instead occurs when a letter string is pronounceable (whether it is a word or not).

Brown noted that the term lexicality cost was used because responses to pseudowords (regularly spelled but meaningless words e.g., kluft) are not associated with the same cost (Brown, Roos-Gilbert, & Carr, 1995), indicating that lexical activation is required. However, more recent work has supported Monsell et al.’s finding that pseudowords are in fact associated with the same cost. Kinoshita et al. (2017) compared Stroop performance on five types of colour-neutral letter strings and incongruent words. They included real words (e.g., *hat*), pronounceable nonwords (or pseudowords; e.g., *hix*), consonant strings (e.g., *hdk*), nonalphabetic symbol strings (e.g., *&@£*), and a row of Xs. They reported that there was a word-likeness or pronounceability gradient.^4^ with real words and pseudowords showing an equal amount of interference (with interference increasing with string length) and more than that produced by the consonant strings. Consonant strings produced more interference than the symbol strings and the row of Xs which did not differ from each other. The absence of the lexicality effect (defined by colour neutral real words producing more interference than pseudowords) was explained by Kinoshita and colleagues as being a consequence of the sub-lexically generated phonology from the pronounceable irrelevant words interfering with the phonetic encoding (articulation planning) of the target colour dimension. Given these findings, Brown’s lexicality cost is perhaps better thought of as a pronounceability cost. If it is information derived directly from the irrelevant letter string that interferes with colour naming when that letter string is pronounceable, then no recourse to task conflict is needed.

#### 4.1.2. Delaying phonetic encoding of the colour name

Just as lexical conflict was conceived of as a cost irrespective of whether the irrelevant word was incongruent or congruent (Brown, 2011), and just as Kalanthroff et al. (2018) proposed that task conflict inhibits all response representations (thereby raising the response threshold) for all word-like stimuli, this pronounceability cost would be incurred irrespective of congruency. Since undertaking the phonological/phonetic encoding of the irrelevant word occurs automatically in the sense that it is obligatory (with both manual and vocal responses), phonetic encoding of the colour name would be delayed. Moreover, since the phonetic information from the irrelevant word is not the needed response, control would likely be needed to prevent it interfering further (especially with a vocal response Stroop task) and control of automatic processes can be effortful (Diede & Bugg, 2017; Entel & Tzelgov, 2020; Parris, Hasshim & Dienes, 2021). This added level of interference in the colour naming process for congruent relative to non-lexical, non-pronounceable letter strings would also add another point at which noise could interact with performance and thereby increase RT variability (see the section above on the description of Kalanthroff et al.’s (2018) model of task conflict).

Supporting evidence for the delaying of the computation of the colour name due to the phonological/phonetic processing of the irrelevant word can be found in some of the research findings we alluded to above. The finding that low frequency words are colour named more slowly than high-frequency words (e.g., Burt, 2002), indicates that generating sub-lexical phonology and subsequent phonetic encoding of pronounceable letter strings delays the phonetic encoding of the colour name. This would lead to slower colour naming times for all pronounceable letter strings. Furthermore, if, as argued above, the mostly non-lexical (heretofore *non-pronounceable string*) context employed in task conflict studies also increases processing of the irrelevant word (see above), this would lead to further delay and a bigger pronounceability cost. This delay is akin to that suggested by the response exclusion hypothesis (Mahon et al., 2007) to explain the word frequency and semantic interference effects in the picture-word interference task. Consistent with this theory, our alternative account is based on the notion that somewhere in the processing stream phonetic encoding or the “production-ready” phonetic code of pronounceable irrelevant words delays the encoding or the production of the colour name.

#### 4.1.3. There is no positive facilitation in a mostly neutral stimulus context so a congruent phonetic code does not facilitate performance

One limitation of this account is that when the irrelevant word is presented in a congruent colour, it does not seem to make sense to argue that the sub-lexically generated phonology interferes with the segment-to-frame association processing in articulation planning; such information should facilitate segment-to-frame association processing. However, as discussed above, positive facilitation is no longer happening given the mostly neutral (pronounceable or non-pronounceable) word context and thus there is something about a mostly neutral context that prevents facilitating information from being utilised (see ‘*Why is there a lack positive facilitation when task conflict control is supposed to be high?’* above). Further, just as lexical conflict was conceived of as a cost irrespective of whether the irrelevant word was incongruent or congruent (Brown, 2011), and just as Kalanthroff et al. (2018) proposed that task conflict inhibits all response representations (thereby raising the response threshold) for all word-like stimuli, this pronounceability cost would be incurred irrespective of congruency given certain experimental conditions. Since undertaking the phonological/phonetic encoding of the irrelevant word occurs automatically in the sense that it is obligatory (with both manual and vocal responses), phonetic encoding of the colour name would be delayed. Moreover, since the phonetic information from the irrelevant word is not the needed response, control would likely be needed to exclude it and prevent it interfering further (especially with a vocal response Stroop task) and control of automatic processes can be effortful (Diede & Bugg, 2017; Entel & Tzelgov, 2020; Parris, Hasshim & Dienes, 2021).

#### 4.1.4. Phonological processing with manual responses

This account provides a potential explanation as to why the difference between neutral words and repeated letter string trials presented in equal ratios (with no other experimental manipulations and often interpreted as representing task conflict), is harder to observe with manual responses. Kinoshita et al. (2017) reported evidence for sub-lexically generated phonology when participants responded vocally but not when participants responded manually, mirroring the finding that task conflict, when measured as the difference between non-lexical (repeated-letter strings) and neutral word trials, is observed with vocal responses, but not manual responses (Augustinova et al., 2018; Augustinova et al., 2019; Ferrand et al., 2020; Levin & Tzelgov, 2016). Along with Kinoshita et al. (2017), Parris et al. (2019) and Zahedi et al. (2019) have reported data indicating that the difference between manual and vocal responses occurs later in the phonological encoding or articulation planning stage because vocal responses encourage greater phonological encoding than does the manual response (see Van Voorhis & Dark, 1995 for a similar argument). For example, Parris et al. (2019) sought evidence for the use of a serial print-to-speech sub-lexical phonological processing route when using manual and vocal responses by testing for the facilitating effects of phonological overlap between the irrelevant word and the colour name at the initial and final phoneme positions (e.g., *row* in red vs. *bad* in red). The results showed phoneme overlap leads to facilitation with both response modes, but a significantly larger effect with vocal responses. This suggests that the significant difference in mean colour-naming latencies between colour-neutral words and repeated letter-strings only with vocal responses (Augustinova et al., 2018; Augustinova et al., 2019; Levin & Tzelgov, 2016) is a result of the extra phonological processing with vocal responses.

Notably, the fact that most reports of negative facilitation come from studies employing manual responses makes the argument that task conflict is harder to observe with manual responses due to reduced phonological/phonetic processing seem rather self-defeating. Importantly however, it is being argued that observing a difference between neutral word and non-pronounceable letter strings with a manual response is more difficult in a context where the trial types are presented in equal proportions given that phonological processing is reduced with a manual response. When there is a mostly non-pronounceable trial context, control over word processing is potentially much reduced (Kinoshita et al., 2018) and so the differences between trial types that are pronounceable and those that are not is magnified.

### 4.2. Accounting for negative facilitation that is observed without a mostly non-lexical trial context

#### 4.2.1. Negative facilitation in a task-switching context

If negative facilitation reflects task conflict, it would be expected to increase in the task switching context. More specifically, if task set competition were responsible for negative facilitation, it would also be expected that negative facilitation would be larger on task switching trials compared to trials on which participants repeat the same task, since this would be when task conflict is maximised. Rogers and Monsell (1995) reported evidence in support of this.

In their experiments Rogers and Monsell (1995) had participants switch between two intentionally activated, well-established task sets (odd/even and consonant/vowel judgements established via preexperimental training). In their study, a number-letter pair (e.g., G7) was presented in one of four boxes presented on the computer screen. Participants switched between classifying numbers as odd or even and classifying letters as vowels or consonants depending on the location of the number-letter pair in the form box framework (e.g., classify the number if the character pair appeared in either of the top two positions and the letter if in the bottom two positions). In additional to switch and repeat trials, trials could also be incongruent, congruent or neutral. Incongruent trials were those on which both the letter and the number were associated with opposite responses (i.e., the stimulus ‘G7’ was incongruent because the letter response (consonant) was associated with a left-hand response, and the number response (odd) was associated with a right-hand response. Congruent trials were those on which both attributes were associated with the same response and neutral trials were those on which the irrelevant attribute was associated with neither response (i.e., on a number response trial, the stimulus ‘&7’ could not produce a classification of consonant or vowel). Under these conditions it was shown that responses to incongruent trials were longer and more error prone than those to congruent trials and that responses to congruent trials were longer than those to neutral trials; in other words, negative facilitation was observed. Moreover, this effect of trial type was larger on switch than on repeat trials. Hence, this study provides good evidence that negative facilitation is a measure of task set competition, particularly given that negative facilitation was found to be greater on switch compared to repeat trials. However, this latter effect has not been readily replicated and, moreover, the association between task set competition on switch trials and negative facilitation is not strong.

Using a similar design to that used by Rogers and Monsell (1995), Aron et al. (2004) compared task switching performance on left-frontal (LF) cortex patients, right frontal (RF) cortex patients, and controls. They used the difference between incongruent and congruent trials to measure the effect of task switching on the ability to inhibit the response tendencies activated by the irrelevant task set, and the difference between congruent and neutral stimuli, specifically congruent > neutral or negative facilitation, as an index of the ability to inhibit the irrelevant task set. They reported that LF patients showed greater negative facilitation on switch trials compared to controls, indicating poor control over activation of the irrelevant task set. Furthermore, they showed that, in contrast, RF patients had a greater incongruent – congruent trial difference, indicating an impairment in the ability to inhibit S-R associations which they say were activated by the irrelevant task set.

Interesting, as well as showing greater response inhibition impairment, the RF group in Aron et al.’s study showed a generally larger switch cost than controls. However, in contrast to the control group, the RF group did not show a negative facilitation effect. In fact, in most conditions the RF group showed positive facilitation (Congruent < Neutral or C < N). If negative facilitation represents greater exogenous activation of the irrelevant task set, then a smaller C-N must mean that RF patients had greater control over the exogenous activation of the irrelevant task set. But this is not consistent with the finding that the RF group showed a greater switch cost compared to controls. And while the RF group’s increase in switch cost could be argued to be attributable mainly to the incongruent trials, it is notable that the LF group showed a much-increased general switch cost relative to controls in one condition (short RSI) without a concomitant increase in negative facilitation. These findings are not consistent with the notion that negative facilitation increases as task set competition increases.

Using a cued task switching paradigm (both studies mentioned above employed the alternating-runs task switching paradigm), Steinhauser and Hübner (2007) questioned whether the increased difference between congruent and neutral trials on switch trials was due to increased task conflict or to the increased salience of the distractor after a task switch. They argued that given that the stimulus that currently defines the distractor defined the target on the previous trial, the capability of the distractor to capture attention was enhanced. Consistently, they showed that negative facilitation was not modulated because of competition between task sets per se (task switching), but rather because the salience of the distractors was increased. The authors interpreted their finding as showing that negative facilitation increases only when the salience of the irrelevant dimension is increased. This could be interpreted as showing that the task switch led to greater informational, not task, conflict. Nevertheless, it is important to note that Steinhauser and Hübner (2007) were arguing that distractor salience modulates stimulus-induced *task conflict*. In other words, the salience of the distractor is important only because of the association between the distractor and its task set according to the authors of the study.

Task switching studies that have employed the Stroop task also pose problems for the notion that negative facilitation reflects task set competition. Recall that Kalanthroff and Henik (2014) reported significant negative facilitation only when CTI was 0ms (but not when CTO was 300ms or 1500ms). In other words, negative facilitation was larger in their experiments when task set competition was greater (see also Aron et al., 2004). However, in contrast to Rogers and Monsell (1995), negative facilitation did not differ between switch and repeat trials. Similarly, Aarts et al. (2009) reported negative facilitation in a task-switching context using an arrow-word Stroop-like task but again the magnitude of negative facilitation did not differ between switch and repeat trials indicating that when task conflict is maximised (on switch trials), negative facilitation is not modulated – and in fact, if anything negative facilitation was smaller on switch trials in Aarts et al.’s study (see their Figure 2 p2093).

Perhaps more problematic for the task conflict account of negative facilitation is a study by Steinhauser and Hübner (2009). Like Kalanthroff and Henik (2014), Steinhauser and Hübner manipulated task conflict control by combining the colour-word Stroop task with a task-switching paradigm. Their study consisted of four Stroop conditions which they referred to as: identity trials (standard congruent trials); congruent trials (incongruent trials but where the incongruent word provides evidence towards the same response button as the colour name – also known as same-response trials); incongruent trials (standard incongruent trials where the irrelevant word provides evidence towards a different response key) and univalent trials (repeated-letter string trials). Despite their non-standard nomenclature (which has led to their result being misinterpreted as showing evidence for negative facilitation – see both Kalanthroff & Henik, 2014 and Parris et al., 2022) it is clear from their Figure 2 that switching trials did not produce negative facilitation. Whilst the authors did report that task switching increased what they called the bivalency cost, which was the average RT for bivalent stimuli (incongruent and congruent trials) minus the RT for univalent stimuli (repeated-letter string trials), this appears to be entirely driven by an increase in response times to incongruent stimuli.

Unlike Kalanthroff and Henik (2014), Steinhauser and Hübner (2009) did not manipulate CTI and maintained a CTI of 1200ms across all of their experiments, which means their results are actually consistent with Kalanthroff and Henik’s study in showing no negative facilitation at a long CTI. However, Aarts et al. (2009) employed a variable CTI of between 2000ms and 7000ms and still reported negative facilitation. Whilst this could be explained by the fact that Aarts et al. employed the alternating-runs, predictable switching paradigm, whereas Kalanthroff and Henik (2014) and Steinhauser and Hübner (2009) employed the cued task-switching paradigm, the predictable switching context is more akin to having a longer CTI than a 0ms CTI, indicating that the effect of CTI on negative facilitation is somewhat inconsistent across studies.

Despite these inconsistencies it is noteworthy that both Kalanthroff and Henik (2014) and Aarts et al. (2009) reported negative facilitation in the Stroop task despite not employing a trial-type proportion manipulation (there were equal proportions of incongruent, congruent and repeated-letter trials). Kalanthroff and Henik (2014) argued that the fact that switching did not affect negative facilitation indicated that negative facilitation could be attributed to reduced long-term, proactive control in task-switching contexts. Indeed, the results from most of the above studies indicates that in task-switching studies, negative facilitation results from mixing switch and repeat trials, rather than being related to switch trials. To test this possibility, future studies could compare the magnitude of negative facilitation in task-repetition trials vs. single-task trials akin to Shahar and Meiran (2015; who did not include a neutral trial to enable calculation of negative facilitation). Moreover, future research should explore the role of distractor salience in producing negative facilitation and how this relates to both negative facilitation and informational conflict. For now, however, the fact that across most studies, negative facilitation is not maximised when task set competition is maximised, represents a challenge to the task conflict account of negative facilitation. Under our alternative account, the relationship between mixing switch and repeat trials and negative facilitation is explained as reduced proactive control over informational conflict due to the increased salience of the irrelevant dimension.

#### 4.2.2. Negative facilitation in the Stop-Signal task context

When accounting for negative facilitation that is observed in the Stop-signal task we start by reiterating one of the challenges to the task conflict account noted above: despite the authors sometimes referring to the behavioural marker of task conflict as reverse facilitation, positive facilitation is largely absent in experiments or conditions when it should be present (under the task conflict account). In Kalanthroff et al.’s (2013b) study positive facilitation was small (13ms), but significant in the “go” response trials of their Experiment 1. In Experiment 2 in which the repeated-letter trials were replaced by colour-neutral words (and thus in which task conflict should not be present) positive facilitation was not significant, and neither was it in Experiment 3 which used both repeated-letter and neutral words baselines. Thus, as with a mostly neutral trial context (words or letter strings) there is something about the stop-signal task context that does not favour positive facilitation, despite the trial types being presented in equal proportions.

Under the alternative account, the reason there is negative facilitation on erroneous stop-signal response trials (ESSR) trials is because this is when informational conflict control is reduced and S-R associations can therefore be more influential on performance. Conversely, the reason there is no negative facilitation on go response trials is because informational conflict control is sufficient. Evidence for reduced control over informational conflict would be represented by more Stroop interference on the ESSR trials than on the go response trials. In Kalanthroff et al. (2013b)’s Experiment 1 Stroop interference (incongruent – repeated-letter string trials) goes up from 25ms on go response trials to 36ms on ESSR trials. This increase is not seen in Experiment 2 when the non-lexical trials were replaced with neutral word trials. More convincingly, in the more powerful Experiment 3 interference goes up from 18ms on go response trials to 59ms on ESSR trials. Interestingly, if you look at the effect on interference related to pronounceability (word neutral – repeated-letter string trials) in Experiment 3, interference goes up from 1ms to 60ms, indicating the effect seen on standard Stroop interference is driven entirely by pronounceability and indeed neutral word and incongruent word trial RTs do not differ.

One limitation of our alternative account of the data is that it would predict that the difference between neutral word and incongruent trial RTs in their Experiment 3 should also have been larger because the semantic and response conflict experienced on incongruent trials is a form of informational conflict. This is not what was observed. We would argue that incongruent and neutral word trial RTs would not differ at this point because response and semantic conflict have not yet had the chance to impair performance – and interference is likely based entirely on phonological conflict. In recent work (Martinon et al., submitted), we have shown that response conflict is not robust in the RT distribution until ~450ms post-stimulus onset, and semantic conflict even later (~600ms). The ESSR responses are all between ~410ms and ~480ms. However, it is notable that Kalanthroff et al. did a separate analysis on the fast and slow portions of the response time distribution and showed that their effects replicated even in the slower responses. Nevertheless, even the slower half of responses were fast (461ms-511ms) and so we would not necessarily expect an effect of response distribution half. In short, the alternative account would not expect a relationship between ESSR responses and informational conflict when informational conflict is calculated as incongruent – neutral words. It would however predict a relationship between neutral word – repeated-letter strings given that this measures phonological conflict (pronounceability).

#### 4.2.3. Accounting for Negative facilitation in pupil data

If we are going to provide an alternative account of negative facilitation then we must also address the negative facilitation reported in the pupil data when no manipulations of trial type proportions are present. Pupil dilation has long been used as a measure of the amount of ongoing processing or effort expenditure (Kahneman & Beatty, 1966; Laeng, Sirois & Gredebäck, 2012). Indeed, Hershman and colleagues (2021) argued that stimuli that are more abstract and meaningless (such as a colour patch or an abstract shape) require less mental effort to process and therefore produce less task conflict. The invocation of mental effort and its relationship to task conflict lays the groundwork for an alternative account of the data: pupil dilation might simply reflect processing efficiency. Colour patches do not require much effort to process, so the associated pupil sizes would be relatively small. Words are processed at various levels (orthographic, phonological/phonetic, and semantic) and therefore require more effort and time to process. Pronounceable letter strings also lead to more processing and thus more effort than non-pronounceable letter strings which are simpler to process. When words are less frequent it takes longer, and presumably more effort, for their associated representations to be activated. And this process apparently delays colour naming in the Stroop task. Moreover, the outputs of this obligatory process of grapheme-to-phoneme conversion and the phonetic processing would need to be controlled, and control over automatic processing is effortful (Diede & Bugg, 2017; Entel & Tzelgov, 2020; Parris, Hasshim & Dienes, 2021). Here again then, task conflict need not be invoked; the divergence in pupil sizes might merely reflect: 1) the amount of processing that is required for each stimulus type with the greatest amount of effort required for the stimuli with the greatest levels of processing, and/or; 2) the control required to prevent the irrelevant information from further delaying computation of the phonetic code for the colour name. This account can also explain negative facilitation observed in the pupil data in the numerical Stroop task (Hershman, Beckmann, & Henik, 2022) in which the baseline condition was easy-to-process rectangles and in which phonetic codes would still compete on the number trials (i.e., where participants had to respond to the physically larger number and ignore the numerical value e.g., 1 vs. 2).

### 4.3. Predictions

Since we have argued that negative facilitation occurs because repeated-letter trials do not result in phonetic encoding of the irrelevant letter string, which for orthographically-plausible letter strings delays phonetic encoding of the colour name, a direct and testable prediction is that negative facilitation should be different between words that differ in the ease with which they are pronounced, with both vocal and manual (where colour responses are not said aloud) responses, in a mostly repeated-letter trial context. Under the task conflict account in contrast, negative facilitation should remain constant (and around 0ms) because all the stimuli are words and therefore task conflict control is high.

Above we have argued that negative facilitation in pupil data represents differences in cognitive effort, not task conflict. A direct and testable prediction then is that irrelevant real words that are harder to process phonologically (i.e., words differing in pronunciation difficulty, or words differing in word frequency), will result in greater negative facilitation in pupil size data, despite being equated for potential task conflict.

### 4.4. Applying the alternative account to negative facilitation reported in other selection attention paradigms?

So far, we have focused on the Stroop task where most of the work on the role of task conflict in selective attention has been done. In order to generalize our reasoning beyond this task, in what follows we attempt to account for task conflict observed outside of the Stroop task. Again, whenever possible, testable predictions based on the proposed alternative to the task conflict account are outlined.

#### 4.4.1. The Affordances task

The Affordances Task (AT) utilises the fact that some objects trigger an automatic grasping response (e.g., a cup with a handle). It has been shown that classifying such objects is slower and less accurate when the automatic grasping response activates a different hand to the one required to make the correct button press response (*incongruent* condition) compared to when they activate the same hand (*congruent* condition). This affordances effect is a *response conflict* that manifests on incongruent trials between two possible responses. Littman and Kalanthroff (2020) have investigated the possibility that task conflict plays a role in the AT. Littman and Kalanthroff showed that congruent RTs in the Stroop task predicted congruent RTs in the AT when there was a mostly neutral, non-lexical context; in other words, when task conflict control was low in the Stroop task, slower congruent trial performance predicted congruent trial performance in the AT, indicating the presence of task conflict in the AT. However, they did not have a neutral condition in the AT in this study meaning they could not measure negative facilitation in that task. This was rectified in a subsequent study (Littman & Kalanthroff, 2022).

In Littman and Kalanthroff (2022), the authors had participants perform the AT with three conditions: incongruent, congruent and neutral. In an attempt to provide a neutral condition similar to the non-lexical, repeated-letter stimuli in the Stroop task Littman and Kalanthroff employed images of houses that do not have obvious grasping affordances. They showed that RTs to both incongruent and congruent stimuli were longer than to those to the neutral stimuli thereby evidencing negative facilitation and task conflict.

Clearly, this type of negative facilitation is not accounted for by differences in phonological/phonetic encoding of the irrelevant dimension. However, for negative facilitation to be observed, all that is needed is for the neutral stimulus to be responded to more quickly than the congruent stimulus and this could have happened for a number of reasons that do not involve task conflict. For example, in Littman and Kalanthroff (2022), to respond, participants had to classify the stimuli as being in an upright or inverted position. The peaked roof of the houses used as neutral stimuli would seem to facilitate just such a judgement in comparison to making the same judgement for stimuli such as cups.

Furthermore, as explained above, the obligatory grasping response need not be the result of an exogenously activated, competing task set and could instead be the consequence of simple S-R associations. These associations could be all that is required to explain why congruent trials, which have an obligatory grasping response, take longer to respond to than neutral trials that do not. However, there was no manipulation of trial type proportions in the AT when negative facilitation is observed which means the alternative account would then have to account for this given that negative facilitation in the Stroop task is accounted for as a lack of control over word reading resulting from a mostly non-pronounceable trial context (or the task-switching or Stop-Signal task contexts – plus the role of the baseline stimulus).

It is notable that positive facilitation does not seem to occur in the AT (see Littman & Kalanthroff, 2022, for a discussion of this issue), which the alternative account argues is a prerequisite for the observance of negative facilitation (facilitation is never reversed). Even in their Experiment 2 in which Littman and Kalantroff did compare a mostly neutral trial context to a mostly non-neutral trial context, positive facilitation was not observed in the latter. Indeed, the mostly neutral trial condition enhanced negative facilitation, as does its counterpart in the Stroop task – an effect the alternative account would argue resulted from reduced control of S-R associations. Littman and Kalanthroff (2022) argued that positive facilitation is not observed in the AT because either: 1) task conflict is stronger in the AT, or; 2) because along with an obligatory motor response, graspable objects result in elevated motor inhibition (Schulz et al., 2018) indicating that suppressing the grasping response is common (because e.g., cups are not often picked up when they are seen). Thus, whilst congruent trials result in task conflict at the cognitive level, there is more control at the response level. Of course, one would have to consider the relative strength of the affordances effect and concomitant motor inhibition effect, and presumably the former is stronger given the interference effect (incongruent – neutral) seen in the AT. A further consideration is why the enhanced inhibition would not also have an effect on task set conflict.

The alternative account would argue that, as with phonetic codes of irrelevant words, when positive facilitation is not possible, it takes time to inhibit the obligatory motor response for all graspable objects. One prediction that follows from this account is that negative facilitation would be larger for less frequently encountered graspable objects (e.g., saucepans) than for more frequent graspable objects (e.g., cups). Nevertheless, this line of enquiry could prove useful to proponents of task conflict and could further show how the concept of task conflict can account for a variety of findings across an array of experimental conditions.

#### 4.4.2. The Colour-Object Interference Task

The colour-object interference task is a modification of the colour-word Stroop task intended to aid the study of selective attention in children who have not acquired basic reading skills (Cramer, 1967; Prevor & Diamond, 2005). Prevor and Diamond (2005) used the term *colour-object interference* to describe the finding that naming the colour of objects takes longer than naming the colours of abstract forms. La Heij, Boelens, and Kuipers (2010) tested an account of this effect they called the lexical interference account. This account posits that abstract shapes have no lexical labels whereas objects do and thus it is those lexical labels that cause interference on object trials; this is a form of informational conflict. La Heij et al. (2010) argued that the lexical interference account would predict that object names would frequently intrude on colour (or position) naming objects, but noted that this type of error occurred only very infrequently (1.7% of trials) in their study of the effect in children. Moreover, following Monsell et al.’ (2001) reasoning about word frequency and colour naming in the colour-word Stroop task, they argued that the lexical interference account would predict that object naming (as opposed to colour naming) response times for objects should be related to the colour naming times of the same objects; faster named objects should result in slower colour naming. This was also not observed and thus La Heij et al. (2010) argued that their data did not support the lexical interference account. A further experiment supported the finding that colour naming times for objects that were harder vs. easy to name did not differ. La Heij and colleagues therefore argued that their results supported the notion that colour-object interference reflects competition between task sets, not competition between responses (i.e., it was not informational conflict).

We have noted above that Monsell et al.’s (2001) and subsequent studies show that low frequency words interfere more than high frequency words (e.g., Burt, 2002; Dewhurst & Barry, 2006); a finding that has been replicated in the picture-word interference task, a task closely related to the colour-object interference task, with pictures presented with low frequency distractors named slower than those with high frequency distractors (Miozzo & Caramazza, 2003; Dhooge & Hartsuiker, 2010). Given these findings we must consider the strength of evidence for no difference presented by La Heij et al. (2010) between objects that are named faster vs. named slower and objects that are harder to name vs. easier to name.

In their first experiment where La Heij et al. (2010) reported that object naming times did not correlate with colour naming times, their analysis was based on naming of just 28 pictures, and the authors did not report evidence for the null hypothesis. In their second experiment in which they experimentally manipulated the ease of object naming by comparing colour naming times of hard vs. easy items (high vs low familiarity), the authors reported a non-significant object interference effect (27ms, t = 1.6) between the colour naming times of the two sets of items – although they did not report evidence for the null. Moreover, the difference in the colour-object interference effect between the colour naming times of hard-to-name vs. abstract objects (38ms) was only slightly larger, albeit significant (t = 2.8). Thus, the evidence for no difference between hard vs. easy to name items was not strong and further work is needed to confirm this finding. Interestingly however, their analysis excluded any object for which a child provided any verbal label (even when that verbal label was incorrect) indicating that it was not likely that a verbal label, the S-R associated label, interfered in the colour naming process and that the results might be better attributed to task set conflict.

La Heij and Boelens (2011) further tested the task conflict account of the colour-object interference effect and showed that the effect is present in children but not adolescents, a finding that they argued was consistent with an effect resulting from immature cognitive control in the younger children. Furthermore, they showed that the colour-object interference effect is not present when the task is changed from colour naming to sorting by colour, nor was it present when participants were asked to count the number of objects rather than name the colour. The authors concluded that colour-object interference is not the result of a general distracting effect from objects (otherwise it would have been observed in the sorting task) and neither is it dependent on the requirement of verbal responding (otherwise it would have been present when participants had to name the number of objects presented). They argued instead that the effect is best accounted for with reference to task set competition.

In accounting for why tasks such as manual sorting or subitizing do not result in interference La Heij and Boelens (2011) argued that the task set interference account predicts that “the interference effect should be absent in tasks where an identifiable picture does not evoke a prepotent and competing response tendency” (p. 166) and that task sets differ in strength. Notably this explanation is based upon the notion of the response tendency providing the competition, not the task set itself so it is unclear here whether La Heij and Boelens are arguing for task or informational conflict being responsible. Moreover, this account recalls the task set selection problem outlined above: If task sets operate in selective attention tasks, then we have to assume that a task set for manual sorting/subitizing is active, so why do they not provide task set competition? According to La Heij and Boelens, it is because they are not activated strongly enough AND do not activate informational conflict. Thus, under this account interference is not based on task conflict, but on informational conflict produced via the activation of the irrelevant task. But if the informational conflict is prepotent enough to cause interference (and presumably to activate the irrelevant task set?), a question arises as to why the notion of competition between collections of rules and settings is needed.

A further interesting finding reported by La Heij et al. (2010) was that adult participants do not show the colour-object interference effect. This, they argued was due to a fully developed interference control mechanism. The notion here is that interference control is fully functioning in the adults but not the children and that the adults can prevent task conflict, especially when the irrelevant task is not strongly activated. They argue that words are better at activating task sets than are pictures which is why adults do show task conflict in the colour-word Stroop task. However, an alternative interpretation is that words, due to grapheme-to-phoneme correspondence rules, are more readily phonologically processed and thus the basis of all “task set competition” is in fact phonological. We would argue this represents an uncontrolled S-R association in the minds of children whose attentional control capacities are as yet undeveloped.

There is at least one known exception to the finding that only children exhibit colour-object interference. Kalanthroff et al. (2017) reported that adults with obsessive compulsive disorder exhibited impaired performance on the colour-object interference task, but not on the Stop-Signal task, a measure of response inhibition, nor on the arrow-flanker task, a measure of executive abilities not dependent on task conflict control. Moreover, performance on the colour-object interference task correlated with OCD symptom severity. Neither patients with Generalised Anxiety Disorder (GAD) nor a healthy control group showed the same effect.

It is first worth pointing out that in previous work Kalanthroff and colleagues (2013b) have argued that the Stop-Signal task and task conflict share a control mechanism (Kalanthroff, et al., 2013b; see section on ‘Negative facilitation and the Stop-Signal task’ for more details). This work would predict that OCD patients would be impaired on both the Stop-Signal task and the colour-object interference task. Notably, however, when looking at the Stop-Signal (and arrow-flanker task) results, no evidence was present in favour of the null hypotheses, and the general direction of all tasks showed the OCD group as performing worse (longer stop-signal RTs and a larger congruency effect). The interaction term was also not significant (p = .054), and the raw effect size of the colour-object interference effect was small (~10ms) with a Cohen’s d of .43.

Second, we can consider what the correlation between OCD severity and colour-object interference means. As with children studied in La Heij and colleagues work, a larger object interference effect implies greater stimulus-driven behaviour in OCD, and more so in those with more OCD. But does this necessitate an account based on competition between whole task sets? The alternative account would suggest that S-R associations are enough to explain these effects. Task conflict theorists argue that word likeness (processed via S-R associations) are what trigger task sets, but remove task sets from the account and you are left with a series of S-R associations that follow one from the other that might not need a preloaded guide for processing.

We would argue that more work is needed before it can be concluded that these results are due to a deficit in task conflict control, and not simply due to poorer control over informational conflict. The alternative account presented for negative facilitation in the Stroop task is based on grapheme-to-phoneme S-R associations which could not apply here because words are not used. However, an S-R association could also be based on an object being associated with its name. Our alternative account predicts that easy-to-name items would produce less interference than hard-to-name items and that both would take longer to respond to than items that do not have strong S-R associated names (such as abstract shapes). Clearly, La Heij et al. (2010) already ran that study and such predictions were only partially supported. However, we have described the limitations of their findings above and have described research using the closely related picture-word interference task whose results align with our prediction (Miozzo & Caramazza, 2003; Dhooge & Hartsuiker, 2010). Future research could try to replicate the methods employed by La Heij et al. (2010) in children and those of Miozzo and Caramazza (2003) and Dhooge and Hartsuiker (2010) in adults.

Nevertheless, Kalanthroff et al.’s (2017) finding that OCD patients are impaired at the colour-object interference task and not tasks associated with response inhibition is consistent with a selective deficit in task conflict control which the authors likened to a “small-scale” utilisation behaviour. Thus, more supportive evidence in this and other populations showing selective deficits is a useful and promising area for task conflict proponents.

### 4.5. The lexicality problem

A challenge to the alternative account presented here, and perhaps also to the response exclusion hypothesis, is the finding that colour naming does not differ for words and pronounceable non-words (Burt, 2002; Kinoshita et al., 2017; Monsell et al., 2001). It has been shown that reading aloud pseudowords takes longer than it does for words (e.g., Monsell et al. 2001) and thus under the alternative account it should be that colour naming of pseudowords should be slower than those for words.^5^ Furthermore, under the response exclusion account, given slower naming times it would take longer for the pseudoword pronunciation to reach the buffer than it would for a word’s pronunciation. These predictions have not been borne out in the literature, with the bulk of the evidence showing no difference between the two types of stimuli (Burt, 2002; Kinoshita et al., 2017; Monsell et al., 2001). Task conflict theorists would argue that any letter-string that is word-like would produce task conflict and that the magnitude of task conflict would not differ between real words and pseudowords. Hence, the more consistent finding of no difference in colour naming times between these two types of stimuli is consistent with the notion of task conflict and its control contributing to performance on selective attention tasks.

To accommodate this finding in the alternative account, we invoke the notion of the ‘response-relevance criteria’ (Lupker, 1979; Mahon et al., 2007). Whilst the response exclusion hypothesis (Mahon et al., 2007) is acknowledged for its ability to neatly account for the distractor frequency effect and patterns of semantic facilitation and interference in picture-naming, it has come under some criticism, particularly for eschewing lexical selection by competition and the very late locus of selection (see Mulatti & Coltheart, 2012; Roelofs, 2014 etc). Nevertheless, Mulatti and Coltheart (2012) have argued that one of the more promising ideas contained within the approach, which has been presented before in the literature (e.g., Lupker, 1979), is the notion of the ‘response-relevance criteria’, which is the notion that a judgement is made about the relevance of any potential response to the target task. For example, a production-ready response in the articulatory buffer is harder to expunge if it meets the criterion of being semantically related to the target task (e.g., the word ‘cat’ is harder to expunge than the word ‘top’ when the target task is naming animal pictures). Thus, under the response exclusion hypothesis there are two factors that determine picture/colour naming times: 1) the time it takes for the distractor’s pronunciation to reach the buffer; 2) the time it takes the response-relevance criterion to select/reject responses once they are in the buffer (Mahon et al., 2007; Mulatti & Coltheart, 2012). These two factors account for different effects: the distractor frequency effect reflects the speed with which the response to the distractor enters the buffer; the semantic interference effect reflects the speed with which the response is removed from the buffer. We would argue that for pseudowords, whilst the time it takes for the pronunciation to reach the buffer would be longer, it would be easier to decide that a pseudoword is not colour-related than it is to decide a neutral word is not colour related – a process that is perhaps related to imageability (Lupker, 1979). Hence a finding of no effect of lexicality is potentially accounted for by the opposing influence of the need for a response-relevance criterion check.

## 5. Conclusion

In line with the idea of a more pragmatic or perhaps even parsimonious cognitive approach (De Houwer, 2014; 2021), we have proposed that well-established competition/conflict from information derived from S-R processing of the irrelevant dimension could be sufficient to explain at least some of the findings attributed to task conflict. The arguments presented here represent testable and thus falsifiable predictions aimed to stimulate further empirical research. Indeed, only results of cumulative research will provide more definitive answers to whether informational conflict – and in particular its phonological component – in combination with manipulations designed to alter the influence of word processing (e.g., the mostly non-pronounceable letter string context), are sufficient to account for these and future findings.

It is important to emphasize in conclusion that like the notions of conflict adaptation and task set switching, task set conflict is an appealing idea. Indeed, this elegant account – explaining many effects in the literature – has motivated much research. It has revealed effects that might otherwise have been hidden, and has provided important insights into selective attention and cognitive control and their impairment. Moreover, it is certainly the case that whilst we have highlighted weaknesses and challenges to the task conflict account, the burgeoning evidence for it across a wide variety of fields and methods of enquiry (e.g., Obsessive-Compulsive Disorder, RT distributions, pupillometry, fMRI) renders it a potentially powerful approach to understanding the human mind and behaviour.

We have been arguing against the notion that “whole task sets” compete (Monsell et al., 2001) in selective attention tasks. However, it is not clear that sub-sets of task sets could not compete. If a whole task set is, for example, a whole set of S-R associations, then a subset of that whole set, such as grapheme-to-phoneme conversion or conversion of phonological into phonetic codes in the colour-word Stroop task, could be considered sub-sets of the whole, and could compete with the colour naming task set. This would mean that some component of the word reading task set would be competing with the colour naming task set. Selection would then be needed wherever the locus of that sub-set is in processing. This would of course then raise further questions such as what constitutes a subset and whether a task set controller would be required given that it is not whole task sets that are competing.

It is possible that whilst some performance markers that have been taken as evidence for task conflict such as negative facilitation (congruent > non-lexical neutral) and lexical conflict (neutral word > non-lexical neutral), might not represent task conflict, others still might. For example, some studies have revealed that relative to repeated-letter stimuli, stimuli composed of an unpronounceable selection of consonants (e.g., fghdt) produce interference (e.g., Kinoshita et al., 2017). Alphanumerically more complex stimuli such as these might activate a task set for word reading creating task conflict, and then pronounceable letter strings would include additive phonological/phonetic, informational interference, although some consideration would have to be given to the influence of visual complexity (i.e., orthographic similarity to real words) on interference levels. Thus, understanding the role of task set conflict and other forms of conflict often depends on choosing the right baseline, which as previously noted is non-trivial. More recent model-based approaches to measuring the contribution of different processes might prove useful in future endeavours (Evans & Servant, 2022).

It is also apparent that negative facilitation appears under conditions that could be described as representing a high cognitive load (e.g., mixing of switch and repeat trials in task switching, high working memory load, Obsessive-Compulsive Disorder) indicating a vulnerability to task conflict when there is reduced capacity for control. Of course, this could be accounted for under our alternative account as representing poor control of informational conflict, not task set conflict. And whilst there is contradictory evidence for this position (see Entel & Tzelgov, 2020, for evidence that spare working memory capacity is actually required for negative facilitation to be observed), it is possible that negative facilitation is not always the result of the same cause under all conditions. For example, negative facilitation under high cognitive load could be the result of task conflict, but under low cognitive load be due to spare capacity permitting processing phonological/phonetic informational conflict.

Nevertheless, we have taken a critical stance on the task conflict approach as it currently stands, especially with regards to its role in selective attention, and have described what we believe to be challenges to the current iterations of task conflict theory. We have also presented a testable, alternative account of negative facilitation in a variety of contexts, an index that is widely believed to be a marker of task conflict. To the extent that we have highlighted real challenges and presented a reasonable alternative, we will have achieved our aim of encouraging attempts to test and falsify an important conceptual framework in our field.

## Footnotes

It has been suggested that task conflict can be estimated using orthographic (various readable stimuli vs. unreadable shapes) or lexical conflict (real words vs. minimally readable letter strings) measures (Levin & Tzelgov, 2016). However, the orthographic and phonological processing differences between the critical conditions and the baseline conditions in both cases represent informational conflict, and not competition between whole task sets. This is especially true in the case of Levin and Tzelgov’s computation of orthographic and lexical conflict given that they included colour-associated words when calculating their critical condition RT.

MacLeod and McDonald (2000; see also Kane and Engle, 2003) have argued that the facilitating effect of the color-congruent irrelevant word is not true facilitation from any level of processing and is instead the result of ‘inadvertent reading’. That is, on occasional color-congruent trials, participants use only the word dimension to generate a response, meaning that these responses would be 100ms-200ms faster than if they were colour naming (because word reading is that much faster than colour naming). Doing so would equate to the roughly 25ms Stroop facilitation effect observed in most studies and would explain why facilitation is generally smaller than interference. However, the inadvertent reading hypothesis falls short in a number of ways: 1) It is based on the notion that facilitation is generally smaller than interference – a finding that comes from studies using non-lexical baselines and one that has been shown to be unsupported when using lexical baselines (Brown, 2011); 2) It cannot explain facilitation on semantic-associative congruent trials (Augustinova, Parris & Ferrand, 2019) or in cross-language Stroop tasks (Roelofs, 2010) where the facilitating word is not the name of the colour.

The effect of working memory load is unclear since even with a low working memory load, and thus spare working memory capacity, negative facilitation is not present unless participants have been previously exposed to incongruent trials (see Entel & Tzelgov, 2020, Experiment 3). Indeed, as pointed out above, studies investigating the role of working memory load in producing negative facilitation directly contradict each other. Furthermore, negative facilitation has been reported even without exposure to incongruent trials and cueing (Kalanthroff et al., 2013a). Moreover, neither spare working memory capacity, nor exposure to incongruent trials are by themselves, enough to produce negative facilitation i.e., they are both associated with standard Stroop interference and positive facilitation (e.g., Kane & Engle, 2003) indicating that the mostly non-lexical trial context is important when observing negative facilitation.

However, Kinoshita et al. (2017)’s study potentially confounds the orthographic similarity and pronounceability of their pronounceable nonwords (such as HIX) and non-pronounceable consonant nonwords (such as HDK). Indeed, a close examination of their Appendix (pp. 383–384) suggests that the stimuli used in these two conditions are not matched in terms of visual/orthographic similarity to real words. Indeed, pronounceable nonwords (such as HIX) are more similar (orthographically speaking) to real words (e.g., HIM, HIP, HIS, HIT) than are non-pronounceable nonwords (consonants; such as HDK). Thus, more research is needed to establish whether orthographic similarity or pronounceability (or both) is responsible for Kinoshita et al. (2017)’s effect.

We thank Stephen Monsell for pointing this out.
